# 

**Published:** 1889-07

**Authors:** 


### CONTENTS.

***MISCELLANEOUS.*** PAGE

Proceedings of the International Hahnemannian Association, . . 265

Transverse Presentation. Clarence Willard Butler, M. D., . . 275

Fistula in Ano. C. C. Howard, M. D., . . . . . . . 281

The Uses and Abuses of Clinical Records. Edward Cranch, M. D., . 282

Mastitis. Wm. Jefferson Guernsey, M. D., . . . . . . 285

Remedies Affecting the Mammæ. Wm. Jefferson Guernsey, M. D., . 286

Repertory to Labor and After-Pains. John V. Allen, M. D., . . 291

In Praise of Calendula. Alice B. Campbell, M. D., . . . . . 298

Proceedings of the Organon Society of Boston, . . . . . . 299

Bœnninghausen's Croup Powders. A. McNeil, M. D.,, . . . 302

Obituary—Mrs. Wm. A. Hawley, . . . . . . . . . . 305

Sodium Ethylate. George H. Clark, M. D., . . . . . . 306

***BOOK NOTICES AND REVIEWS.***

Lectures on Bright's Disease, by Robert Saundby, M. D., . . 309

The Efficacy of Filters, by Charles G. Currier, M. D., . . . 310

The Eleventh Annual Announcement of College N. Y. Ophthalmic Hospital, . . . . . . . . . . . . . 311

***NOTES AND NOTICES.***

Errata—For Sale—Amer. Pub. Health Asso.—Census Office Notice, . 311

